# Incidence and Transmission Dynamics of *Bordetella pertussis* Infection in Rural and Urban Communities, South Africa, 2016‒2018

**DOI:** 10.3201/eid2902.221125

**Published:** 2023-02

**Authors:** Fahima Moosa, Stefano Tempia, Jackie Kleynhans, Meredith McMorrow, Jocelyn Moyes, Mignon du Plessis, Maimuna Carrim, Florette K. Treurnicht, Orienka Helferscee, Thulisa Mkhencele, Azwifarwi Mathunjwa, Neil A. Martinson, Kathleen Kahn, Limakatso Lebina, Floidy Wafawanaka, Cheryl Cohen, Anne von Gottberg, Nicole Wolter, for the PHIRST Group

**Affiliations:** University of the Witwatersrand, Johannesburg, South Africa (F. Moosa, S. Tempia, J. Moyes, M. du Plessis, M. Carrim, O. Hellferscee, C. Cohen, A. von Gottberg, N. Wolter);; National Institute for Communicable Diseases of the National Health Laboratory Service, Johannesburg (F. Moosa, J. Kleynhans, J. Moyes, M. du Plessis, M. Carrim, F.K. Treurnicht, O. Hellferscee, T. Mkhencele, A. Mathunjwa, C. Cohen, A. von Gottberg, N. Wolter);; Centers for Disease Control and Prevention, Atlanta, Georgia, USA (S. Tempia, M.L McMorrow);; Johns Hopkins University, Baltimore, Maryland, USA (N.A. Martinson);; National Research Foundation, Pretoria, South Africa (N.A. Martinson);; South African Medical Research Council, Cape Town, South Africa (N.A. Martinson, K. Kahn, L. Lebina, K. Mothlaoleng, F. Wafawanaka, A. Mathee);; North-West University, Potchefstroom, South Africa (S. Piketh)

**Keywords:** *Bordetella pertussis*, bacteria, incidence and transmission dynamics, infection, respiratory infections, rural and urban communities, incidence, cohort study, household transmission, colonization, South Africa, pertussis, whooping cough

## Abstract

We conducted 3 prospective cohort studies (2016–2018), enrolling persons from 2 communities in South Africa. Nasopharyngeal swab specimens were collected twice a week from participants. Factors associated with *Bordetella pertussis* incidence, episode duration, and household transmission were determined by using Poisson regression, Weibull accelerated time-failure, and logistic regression hierarchical models, respectively. Among 1,684 participants, 118 episodes of infection were detected in 107 participants (incidence 0.21, 95% CI 0.17–0.25 infections/100 person-weeks). Children <5 years of age who had incomplete vaccination were more likely to have pertussis infection. Episode duration was longer for participants who had higher bacterial loads. Transmission was more likely to occur from male index case-patients and persons who had >7 days infection duration. In both communities, there was high incidence of *B. pertussis* infection and most cases were colonized.

Despite high vaccine coverage with either the whole-cell or acellular vaccine in many countries, the incidence of pertussis has increased globally during the past 20 years (*1**–**4*). Disease increase has been attributed to several factors, including increased awareness by clinicians, more sensitive molecular diagnostic techniques (*2**,**4*), serologic markers for identification of infection in adolescents and adults who are commonly asymptomatic carriers of *Bordetella pertussis* (*1*), pathogen adaptation, or waning immunity (*3**,**5*).

Infection indicates that bacteria are in or on the body and make a person sick. Colonization indicates that bacteria are on the body but do not make a person sick.

In South Africa, pertussis is a notifiable medical condition (*6*). The whole-cell pertussis vaccine was introduced in South Africa in 1950 and was replaced by the acellular pertussis vaccine in April 2009. The vaccine is given to infants at 6, 10, and 14 weeks of age, and a booster dose is given at 18 months of age. In 2019, according to the World Health Organization/United Nations Children’s Fund, coverage for the first dose of the acellular vaccine in South Africa was 84%, and coverage for the third dose was 77% (*7*).

Data on pertussis epidemiology in low- and middle-income countries, particularly in Africa, are lacking; as a result, the epidemiology of the disease is poorly understood (*8*). In South Africa during 2013‒2018, among persons hospitalized because of pneumonia, annual *B. pertussis* incidence was 17 cases/100,000 population, and the highest incidence of disease and most deaths were in children <1 year of age (*9*). In 2018, clusters of pertussis were observed in several provinces in South Africa, and 54% of cases were detected in infants <3 months of age (*10*). Infants <1 year of age were at highest risk for severe pertussis and death. It is essential to understand infection in older children and adults because these age groups are likely sources of transmission to infants (*3*,*5*). This study reports the incidence, factors associated with infection, duration of infection, and transmission dynamics in persons of all ages in an urban community and a rural community in South Africa.

## Materials and Methods

### Study Population

During 2016–2018 (May–October 2016, January–October 2017, and January–October 2018), we conducted a community cohort study in 2 communities in South Africa: 1 rural (Agincourt, Mpumalanga Province) and 1 urban (Klerksdorp, North West Province). We enrolled consenting members of randomly selected households each year. During the household enrollment process, at least 80% of the household members within each household had to consent to be included in the study for a household to be enrolled. A detailed account of the study methods and the cohort profile has been previously reported (*11*,*12*).

### Household Visits

Demographic and baseline health status (including HIV) were collected for all participants at enrollment. Participants were considered HIV infected if they had 1 of the following during the follow-up period: 2 positive rapid HIV test results, evidence of a positive HIV laboratory result, or evidence of receiving antiretroviral treatment. Vaccination status was obtained for children <5 years of age from Road-to-Health Cards, which serve as a child’s formal health record in South Africa. Households were visited twice a week for nasopharyngeal specimen collection and symptom determination by using a structured questionnaire. Symptom data included fever (self-reported or measured tympanic temperature >38°C), cough, difficulty breathing, sore throat, nasal congestion, chest pain, muscle aches, headache, vomiting, or diarrhea. In addition, during 2018, persons who tested PCR positive for *B. pertussis* were retrospectively interviewed (interviews were conducted immediately after a positive PCR result) to confirm if the persons experienced any pertussis-specific symptoms (cough, inspiratory whoop, posttussive vomiting, or apnea) during the infection.

### Specimen Collection and Testing

At each visit, a nasopharyngeal swab specimen was collected and placed in PrimeStore Molecular Transport Medium (Longhorn Vaccines & Diagnostics, https://lhnvd.com) and transported to the laboratory within 48–72 hours for testing. Specimens were received in the laboratory in real-time and then batched for further processing. Total nucleic acids were extracted by using the Roche MagNA Pure 96 Instrument (Roche Diagnostics, https://www.roche.com) and the MP96 DNA and Viral NA SV Kits (Roche Diagnostics). Extracts were tested by using an internally validated IS*481* and human ribonuclease P (RNaseP) duplex real-time PCR. Any specimen that tested positive for IS*481* with a cycle threshold (Ct) value <45 was repeated (from extraction using a fresh aliquot) and retested (2 replicates) by using a second, multitarget real-time PCR targeting *B. pertussis*, *B. parapertussis*, and *B. holmesii* (*13*). A specimen was considered positive for *B. pertussis* if IS*481* or *ptxS1* targets was detected with Ct values <45 in at least 2/3 replicates and negative for hIS*1001* (*B. holmesii*) and pIS*1001* (*B. parapertussis*).

### Study Definitions and Data Analysis

We defined an episode of *B. pertussis* infection as >1 consecutive visits in which *B. pertussis* was detected. A new episode was one that began after a period of at least 12 consecutive visits (i.e., 6 weeks) in which *B. pertussis* was not detected. We determined the incidence of *B. pertussis* infection by dividing the number of PCR-positive episodes by the person-time under observation, expressed as 100 person-weeks. We assessed factors associated with incidence by using Poisson regression accounting for the person-time under observation. For analysis of incidence, we considered all identified episodes of infections, including multiple episodes in the same person.

We estimated the duration of a *B. pertussis* infection as the date of the last positive specimen minus the date of the first positive specimen (within the same episode of infection) plus 2.5 days to account for gaps between visits; values are expressed as mean +SD. We performed analysis of factors associated with the duration of infection (time-to-event outcome) by using accelerated time failure Weibull regression and analysis of *B. pertussis* bacterial loads by using IS*481* Ct as a proxy for bacterial load. We characterized IS*481* bacterial load as follows: Ct <34, higher bacterial load; Ct 34–39, intermediate bacterial load; Ct 40–44, lower bacterial load.

We defined an episode of symptomatic *B. pertussis* infection as illness in a person who had >1 symptoms reported from 1 visit before to 1 visit after the *B. pertussis* episode. Signs and symptoms included fever, cough, difficulty breathing, sore throat, nasal congestion, chest pain, muscle aches, headache, vomiting, and diarrhea. In 2018, we used the pertussis-specific symptom data collected to determine if positive cases could be classified as pertussis by using the South Africa notifiable medical condition pertussis case definition (cough lasting >14 days, or cough of any duration for children <1 year of age, along with >1 of the following symptoms: paroxysms of coughing, inspiratory whoop, posttussive vomiting, and apnea [with or without cyanosis; only for infants <1 year of age]).

A cluster of cases included all *B. pertussis* infections in a single household that occurred within an interval of <7 days. The index case was the first person testing positive within a cluster. We estimated cluster duration as the time from the first day of positivity of the first person in a cluster to the last day of positivity of the last person. We defined household cumulative infection risk (HCIR) as the proportion of subsequent infections within a household after the first PCR-positive case and evaluated HCIR among all households in which >1 household member had *B. pertussis* infection. The formula for HCIR was the number of infected persons within a household (excluding the index case) divided by number of enrolled persons living within the household. We excluded households that had coprimary cases from the HCIR analysis. We defined crowding as >2 persons sharing a sleeping room within the household.

We used logistic regression to analyze factors associated with symptomatic fraction and HCIR. Pertussis vaccine status was based on number of vaccine doses received by age. For all analyses, we accounted for clustering by study site and households within site by using hierarchical mixed effect models. For the multivariable models, we assessed all variables that were significant at p<0.2 by univariate analysis and removed nonsignificant factors (p>0.05) by using manual backward elimination. We set statistical significance at p<0.05 and performed all statistical analyses in Stata version 14.1 (StataCorp LP, https://www.stata.com).

### Ethics

Ethics approval for the Prospective Household cohort study of Influenza, Respiratory Syncytial virus, and other respiratory pathogens community burden and Transmission dynamics in South Africa (PHIRST) was obtained from the University of the Witwatersrand Human Research Ethics Committee, South Africa (protocol no. 150808, C.C.). Written informed consent was obtained from all enrolled persons, or parents/guardians in the case of minors. A separate ethics application for the pertussis component of the PHIRST was obtained from the University of the Witwatersrand Human Research Ethics Committee, South Africa (protocol no. M210676, F.M.).

## Results

### Study Population

During 2016–2018, a total of 1,684 persons from 327 households were enrolled: 542 persons in 2016, 577 in 2017, and 565 in 2018 (Table 1), as described (*12*). Overall, 16.6% (279/1,684) of the participants were <5 years of age, of whom 97.3% (214/220) were fully vaccinated for age with the acellular *B. pertussis* vaccine. HIV prevalence for the cohort was 15.3% (249/1,628).

**Table 1:** Characteristics for 1,684 persons enrolled in the PHIRST, South Africa, 2016–2018*

Characteristic	No. positive/no. tested (%)		
	Overall	Rural	Urban
Year			
2016	542/1,684 (32.2)	280/849 (32.9)	262/835 (31.4)
2017	577/1,684 (34.3)	289/849 (34.0)	288/835 (34.5)
2018	565/1,684 (33.6)	280/849 (32.9)	285/835 (34.1)
Sex			
M	675/1,684 (40.1)	316/849 (37.2)	359/835 (42.9)
F	1,009/1,684 (59.9)	533/849 (62.8)	479/835 (57.1)
Age group, y			
<1	36/1,684 (2.1)	15/849 (1.8)	21/835 (2.5)
1–4	243/1,684 (14.4)	156/849 (18.4)	87/835 (10.4)
5–14	547/1,684 (32.5)	309/849 (36.4)	238/835 (28.5)
15–24	273/1,684 (16.2)	124/849 (14.6)	149/835 (17.8)
25–44	317/1,684 (18.8)	141/849 (16.6)	176/835 (21.1)
45–64	195/1,684 (11.6)	74/849 (8.7)	121/835 (14.5)
>65	73/1,684 (4.3)	30/849 (3.5)	43/835 (5.2)
HIV status			
Negative	1,379/1,628 (84.7)	715/832 (85.9)	664/796 (83.4)
Positive	249/1,628 (15.3)	117/832 (14.1)	132/796 (16.6)
Nutritional status†			
Underweight	131/1,676 (7.8)	55/849 (6.5)	76/827 (9.2)
Normal	993/1,676 (59.3)	552/849 (65.0)	441/827 (53.3)
Overweight	265/1,676 (15·8)	123/849 (14.5)	142/827 (17.2)
Obese	287/1,676 (17.1)	119/849 (14.0)	168/827 (20.3)
Underlying illness‡			
No	1,634/1,684 (97.0)	844/849 (99.4)	790/835 (94.6)
Yes	50/1,684 (3.0)	5/849 (0.6)	45/835 (5.4)
Pertussis vaccination§			
Incomplete	6/220 (2.7)	3/128 (2.3)	3/92 (3.3)
Fully vaccinated	214/220 (97.3)	125/128 (97.7)	89/92 (96.7)
Crowding¶			
No	738/1,684 (43.8)	361/849 (42.5)	377/835 (45.2)
Yes	946/1,684 (56.2)	488/849 (57.5)	458/835 (54.9)

*PHIRST, Prospective Household cohort study of Influenza, Respiratory Syncytial virus, and other respiratory pathogens community burden and Transmission dynamics in South Africa. †Nutritional status is based on a person’s body mass index (BMI). We defined BMI categories as follows: underweight, age <18 y weight for age or BMI <‒2 SDs of World Health Organization (WHO) Child Growth Standards; age >18 y BMI <18.5 kg/m^2^; overweight, age <18 y BMI >+1 and ≤+2 SD of the WHO growth standards, age ≥18 y BMI ≥25 and <30kg/m^2^; obese, age <18 y BMI >+2 SD of WHO growth standards, age ≥18 y BMI >30 kg/m^2^. ‡Defined as self-reported history of asthma, lung disease, heart disease, stroke, spinal cord injury, epilepsy, organ transplant, immunosuppressive therapy, organ transplantation, cancer, liver disease, renal disease, or diabetes. §Collected only for children <5 y of age. ¶More than 2 persons in a household sleeping in the same room.

### Specimen-Level Data

There were 122,133 possible individual follow-up visits, from which 105,687 (86.5%) nasopharyngeal swab specimens were collected and tested for *Bordetella* species (Appendix Figures 1‒3). Of the specimens tested, 276 (0.3%) were PCR positive for *B. pertussis* and 413 (0.4%) were PCR positive for *B. holmesii*. *B. parapertussis* was not detected. Of the specimens positive for *B. pertussis*, 20.3% (56/276) were positive for both IS*481* and ptx*S1*, and 79.7% (220/276) were positive for IS*481* only.

### Individual-Level Data

#### Incidence and Factors Associated with Incidence

Among 1,684 study participants, 118 episodes of *B. pertussis* infection were detected in 107 participants; 11 persons had 2 episodes of *B. pertussis* infection. There were no demographic/clinical differences observed between persons who had 1 or 2 episodes of infection. The incidence of *B. pertussis* infection was 0.21 (95% CI 0.17–0.25)/100 person-weeks (Table 2). The highest incidence of *B. pertussis* infection was observed in persons 5–14 years of age (0.27 [95% CI 0.20–0.35]/100 person-weeks). Children <5 years of age who had incomplete vaccination for age were more likely to be infected with *B. pertussis* than were vaccinated children (adjusted incidence rate ratio 4.48, 95% CI 1.38–14.59). Among the 36 enrolled infants, 5 *B. pertussis* infections were identified in those 9–11 months of age. Of those infants, 4 were fully vaccinated for age; 1 infant (incomplete vaccination) had symptoms (runny nose) during the *B. pertussis* infection.

**Table 2:** Incidence rate and factors associated with *Bordetella pertussis* incidence for 1,684 participants in the PHIRST study of respiratory infections, South Africa, 2016–2018*

Characteristic	*B. pertussis* incidence (95% CI)	Univariate analysis			Multivariable analysis	
		Incidence rate ratio (95% CI)	p value		Adjusted incidence rate ratio (95% CI)	p value
Overall	0.2 (0.17–0.25)	NA	NA		NA	NA
Year						
2016	0.25 (0.17–0.35)	Referent			NA	NA
2017	0.13 (0.09–0.19)	0.53 (0.17–1.63)	0.27		NA	NA
2018	0.26 (0.20–0.33)	1.04 (0.44–2.49)	0.92		NA	NA
Site						
Rural	0.17 (0.13–0.22)	Referent			NA	NA
Urban	0.24 (0.19–0.31)	1.5 (0.77–2.96)	0.23		NA	NA
Sex						
M	0.23 (0.17–0.30)	Referent			NA	NA
F	0.19 (0.15–0.24)	0.83 (0.58–1.19)	0.31		NA	NA
Age group, y						
<5	0.21 (0.14–0.33)	1.41 (0.69–2.90)	0.37		1.62 (0.56–4.65)	0.37
5–14	0.27 (0.20–0.35)	1.76 (0.96–3.21)	0.09		1.73 (0.91‒3.31)	0.09
15–44	0.17 (0.12–0.24)	1.12 (0.62–2.04)	0.78		1.09 (0.59–2.00)	0.78
>45	0.15 (0.09–0.25)	Referent			Referent	
HIV status						
Uninfected	0.22 (0.18–0.27)	Referent			NA	NA
Infected	0.14 (0.08–0.25)	0.64 (0.36–1.11)	0.11		NA	NA
Nutritional status†						
Underweight	0·20 (0.10–0.38)	0.68 (0.28–1.66)	0.39		NA	NA
Normal	0.23 (0.18–0.28)	Referent			NA	NA
Overweight/obese	0.30 (0.21–0.43)	0.76 (0.52–1.13)	0.18		NA	NA
Underlying illness‡						
No	0.20 (0.17–0.24)	Referent			NA	NA
Yes	0.36 (0.16–0.80)	1.78 (0.68–4.70)	0.24		NA	NA
Pertussis vaccination§						
Incomplete	0.91 (0.23–3.64)	4.89 (1.47–16.02)	0.01		**4.48 (1.38–14.59)**	**0.01**
Fully vaccinated	0.19 (0.11–0.32)	Referent			Referent	
Unknown	0.20 (0.17–0.25)	1.08 (0.61–1.91)	0.78		1.32 (0.42–4.13)	0.64
Crowding¶						
No	0.15 (0.11–0.21)	Referent			Referent	
Yes	0.25 (0.20–0.31)	1.63 (0.86–3.08)	0.13		1.53 (0.78–2.97)	0.21

*Bold indicates statistical significance. Variables adjusted for in final model were age group, pertussis vaccination, and crowding. NA, not applicable; PHIRST, Prospective Household cohort study of Influenza, Respiratory Syncytial virus, and other respiratory pathogens community burden and Transmission dynamics in South Africa. †Nutritional status is based on a person’s body mass index (BMI). We defined BMI categories as follows: underweight, age <18 y weight for age or BMI <‒2 SDs of World Health Organization (WHO) Child Growth Standards; age >18 y BMI <18.5 kg/m^2^; overweight, age <18 y BMI >+1 and ≤+2 SD of the WHO growth standards, age ≥18 y BMI ≥25 and <30 kg/m^2^; obese, age <18 y BMI >+2 SD of WHO growth standards, age ≥18 y BMI >30 kg/m^2^. ‡Defined as self-reported history of asthma, lung disease, heart disease, stroke, spinal cord injury, epilepsy, organ transplant, immunosuppressive therapy, organ transplantation, cancer, liver disease, renal disease, or diabetes. §Collected only for children <5 y of age· Vaccine status calculate based on number of doses of vaccine received by age. ¶More than 2 persons in a household sleeping in the same room.

#### Duration of PCR-Positive *B. pertussis* Infection

The mean ± SD duration of *B. pertussis* infection was 12.0 +19.1 days. The duration of *B. pertussis* infection was longer when >2 symptoms were reported (adjusted hazard ratio 0.26, 95% CI 0.18–0.67) (Table 3) when compared with colonized persons. Duration of infection was longer for participants who had higher and intermediate bacterial loads (Ct values <34 and 35–39) when compared with persons who had lower bacterial loads (Ct values 40–44; adjusted hazard ratio for Ct <34, was 0.16 [95% CI 0.06–0.44] and for wCt 35–39 was 0.41 [95% CI 0.22–0.74]).

**Table 3:** Factors associated with PCR-positive *Bordetella pertussis* infection duration for 118 persons participants in the PHIRST study of respiratory infections, South Africa, 2016–2018*

Characteristic	*B. pertussis* mean ± SD infection duration, d	Univariate analysis			Multivariable analysis	
		Hazard ratio (95% CI)	p value		Adjusted hazard ratio (95% CI)	p value
Sex						
M	11.9 ± 21.1	Referent			NA	NA
F	14.1 ± 18.8	0.77 (0.45–1.31)	0.34		NA	NA
Age group, y						
<5	6.3 ± 6·2	1.87 (0.84–4.20)	0.13		2.20 (0.87–5.58)	0.09
5–14	12.3 ± 14.5	1.09 (0.59–2.02)	0.78		1.16 (0.58–2.33)	0.67
15–44	15.4 ± 20.8	1.16 (0.50–2.67)	0.73		2.07 (0.81–5.27)	0.13
>45	22.4 ± 38.8	Referent			Referent	
HIV status						
Uninfected	13.8 ± 20.7	Referent			NA	NA
Infected	9.3 ± 10.2	1.06 (0.50–2.26)	0.88		NA	NA
Nutritional status†						
Underweight	4.4 ± 2.7	2.78 (0.98–8.00)	0.06		NA	NA
Normal	13.0 ± 18.7	Referent			NA	NA
Overweight/obese	16.2 ± 25.3	0.44 (0.71–2.17)	0.44		NA	NA
Underlying illness‡						
No	12.4 ± 18.7	Referent			NA	NA
Yes	28.2 ± 34.9	0.80 (0.27–2.33)	0.68		NA	NA
Pertussis vaccination§						
Incomplete	3.0 ± 0.0	2.44 (0.33–17.82)	0.38		NA	NA
Fully vaccinated	6.7 ± 6.6	Referent			NA	NA
Unknown	14.4 ± 21.1	0.63 (0.29–1.36)	0.24		NA	NA
Symptoms						
None	9.3 ± 14.1	Referent			Referent	
1	22.2 ± 20.3	0.62 (0.19–2.04)	0.44		0.39 (0.18–1.62)	0.19
>2	11.1 ± 10.2	0.38 (0.16–0.93)	0.03		**0.26 (0.8–0.67)**	**0.006**
IS*481* Ct category¶						
*<*34	17.0 ± 11.6	0.17 (0.06–0.45)	<0.001		**0.16 (0.06–0.44)**	**0.0001**
35–39	13.5 ± 20.7	0.43 (0.24–0.76)	0.004		**0.41 (0.22–0.74)**	**0.003**
40–44	11.1 ± 20.7	Referent			Referent	

*Bold indicates statistical significance. Variables adjusted for in final model: age group, presence/absence of symptoms, and IS*481* Ct category. Ct, cycle threshold; IS, insertion sequence; NA, not applicable; PHIRST, Prospective Household cohort study of Influenza, Respiratory Syncytial virus, and other respiratory pathogens community burden and Transmission dynamics in South Africa. †Nutritional status is based on a person’s body mass index (BMI). We defined BMI categories as follows: underweight, age <18 y weight for age or BMI <‒2 SDs World Health Organization (WHO) Child Growth Standards; age >18 y BMI <18.5 kg/m^2^; overweight, age <18 y BMI >+1 and ≤+2 SD of the WHO growth standards, age ≥18 y BMI ≥25 and <30kg/m^2^; obese, age <18 y BMI >+2 SD of WHO growth standards, age ≥18 y BMI >30 kg/m^2^. ‡Defined as self-reported history of asthma, lung disease, heart disease, stroke, spinal cord injury, epilepsy, organ transplant, immunosuppressive therapy, organ transplantation, cancer, liver disease, renal disease, or diabetes. §Collected only for children <5 y of age· Vaccine status calculate based on number of doses of vaccine received by age. ¶Used as a proxy for bacterial load.

#### Factors Associated with Symptomatic *B. pertussis* Infection

Of the 107 persons positive for *B. pertussis*, 34 (31.8%) reported symptoms during any given episode of infection (Table 4). Common respiratory signs and symptoms reported during an episode of *B. pertussis* infection were runny nose (55.9%, 19/34), cough (82.4%, 28/34), fever (29.4%, 10/34), and sore throat (14.7%, 5/34). Other, less commonly reported, symptoms were muscle aches, difficulty breathing, chest pain, and vomiting. In 2018, a total of 47.1% (24/51) of persons testing positive for *B. pertussis* were retrospectively interviewed (Appendix Table). A total of 83% (20/24) of these persons reported >1 symptom consistent with pertussis (cough, inspiratory whoop, posttussive vomiting, or apnea) and 60.0% (12/20) of symptomatic persons met the pertussis clinical case definition. Among the symptomatic *B. pertussis*–positive persons <5 years of age, 67.0% (4/6) were fully vaccinated for age. Persons positive for *B. pertussis* were 4 times more likely to have symptoms if they were infected for >7 days than persons infected for <7 days (adjusted odds ratio [aOR] 4.12, 95% CI 1.70–9.98) (Table 4).

**Table 4:** Factors associated with symptomatic *Bordetella pertussis* infection for 107 persons participants in the PHIRST study of respiratory infections, South Africa, 2016–2018*

Characteristic	*B. pertussis* symptomatic, no. positive/no. tested (%)	Univariate analysis			Multivariable analysis	
		Odds ratio (95% CI)	p value		Adjusted odds ratio (95% CI)	p value
Year						
2016	6/33 (18.2)	Referent			NA	NA
2017	2/29 (31.0)	2.03 (0.62–6.62)	0.24		NA	NA
2018	19/56 (33.9)	2.31 (0.81–6.56)	0.12		NA	NA
Site						
Rural	13/48 (27.1)	Referent			NA	NA
Urban	21/70 (30.0)	1.15 (0.51–2.61)	0.73		NA	NA
Sex						
M	10/52 (19.2)	Referent			NA	NA
F	24/66 (36.4)	2.40 (1.02–5.63)	0.04		NA	NA
Age group, y						
<5	4/20 (20.0)	0.25 (0.05–1.14)	0.07		0.33 (0.06–1.69)	0.18
5–14	15/56 (26.8)	0.37 (0.11–1.22)	0.10		0.41 (0.11–1.54)	0.19
15–44	8/28 (28.6)	0.40 (0.11–1.51)	0.18		0.28 (0.06–1.22)	0.09
>45	7/14 (50.0)	Referent			Referent	
HIV status						
Uninfected	28/104 (26.9)	Referent			Referent	
Infected	6/12 (50.0)	2.71 (0.81–9.11)	0.11		3.37 (0.77–14.56)	0.10
Nutritional status†						
Underweight	0/9 (0.0)	0.15 (0.01–2.65)	0.19		NA	NA
Normal	20/77 (25.9)	Referent			NA	NA
Overweight/obese	13/28 (46.4)	2.44 (1.00–5.93)	0.05		NA	NA
Underlying illness‡						
No	31/112 (27.7)	Referent			NA	NA
Yes	3/6 (50.0)	2.61 (0.50–13.65)	0.26		NA	NA
Pertussis vaccination§						
Incomplete	0/2 (0.0)	0.47 (0.02–11.81)	0.64		NA	NA
Fully vaccinated	4/14 (28.6)	Referent			NA	NA
Unknown	30/102 (29.4)	0.98 (0.30–3.20)	0.98		NA	NA
IS*481* Ct category¶						
<34	6/15 (40.0)	3.05 (0.82–11.38)	0.09		NA	NA
35–39	21/64 (32.8)	2.23 (0.85–5.89)	0.11		NA	NA
40–44	7/39 (17.9)	Referent			NA	NA
Episode duration, d						
<7	14/75 (18.7)	Referent			Referent	
>7	20/43 (46.5)	3.79 (1.64–8.73)	0.002		**4.12 (1.70–9.98)**	**0.002**
Smoking, >15 y of age						
No	14/37 (37.8)	Referent			NA	NA
Yes	5/16 (31.3)	0.75 (0.21–2.60)	0.65		NA	NA

*Bold indicates statistical significance. Variables adjusted for in final model: age group, HIV status, and episode duration. Ct, cycle threshold; IS, insertion sequence; NA, not applicable; PHIRST, Prospective Household cohort study of Influenza, Respiratory Syncytial virus, and other respiratory pathogens community burden and Transmission dynamics in South Africa. †Nutritional status is based on a person’s body mass index (BMI). We defined BMI categories as follows: underweight, age <18 y weight for age or BMI <‒2 SDs World Health Organization (WHO) Child Growth Standards; age >18 y BMI <18.5 kg/m^2^; overweight, age <18 y BMI >+1 and ≤+2 SD of the WHO growth standards, age ≥18 y BMI ≥25 and <30kg/m^2^; obese, age <18 y BMI >+2 SD of WHO growth standards, age ≥18 y BMI >30 kg/m^2^. ‡Defined as self-reported history of asthma, lung disease, heart disease, stroke, spinal cord injury, epilepsy, organ transplant, immunosuppressive therapy, organ transplantation, cancer, liver disease, renal disease, or diabetes. §Collected only for children <5 y of age. Vaccine status calculate based on number of doses of vaccine received by age. ¶Used as a proxy for bacterial load.

#### Household Cumulative Infection Risk

The overall household cumulative infection risk was 14.4% (43/298 susceptible exposed persons). Transmission was more likely to occur from male index case-patients than female index case-patients (aOR 12.20, 95% CI 1.57–94.96) and persons who had >7 days episode duration than <7 days (aOR 24.79, 95% CI 2.74–224.30) (Table 5). Within households with confirmed *B. pertussis* transmission, 38.8% (14/36) of index case-patients transmitting to household contacts were colonized.

**Table 5:** Factors associated with household cumulative infection risk for 118 persons, PHIRST, South Africa, 2016–2018*

Characteristic	*B. pertussis* HCIR, no. positive/no. tested (%)	Univariate analysis			Multivariable analysis	
		Odds ratio (95% CI)	p value		Odds ratio (95% CI)	p value
Characteristics of index case-patient						
Sex						
M	30/143 (20.9)	16.81 (1.43–197.69)	0.025		**12.20 (1.57–94.96)**	**0.02**
F	6/150 (4.0)	Referent			Referent	
Age group, y						
<5	2/43 (4.7)	0.05 (0.0003–7.40)	0.24		NA	NA
5–14	27/182 (14.8)	0.38 (0.02–7.59)	0.53		NA	NA
15–44	1/42 (2.4)	0.03 (0.0001–14.52)	0.27		NA	NA
>45	6/22 (27.3)	Referent			NA	NA
IS*481* Ct category†						
<34	16/57 (28.1)	Referent			NA	NA
35–39	11/138 (7.9)	0.35 (0.02–6.17)	0.47		NA	NA
40–44	9/98 (9.2)	0.43 (0.02–8.87)	0.59		NA	NA
Episode duration, d						
<7	12/216 (5.7)	Referent			Referent	
>7	24/77 (31.2)	40.84 (3.37–495.19)	0.004		**24.79 (2.74–224.30)**	**0.004**
Characteristics of household contacts						
Sex						
M	15/110 (13.6)	Referent			NA	NA
F	21/183 (11.5)	0.65 (0.22–1.93)	0.44		NA	NA
Age group, y						
<5	8/50 (16.0)	6.60 (1.22–35.64)	0.03		NA	NA
5–14	11/88 (12.5)	2.10 (0.51–8.73)	0.31		NA	NA
15–44	13/106 (12.3)	2.32 (0.30–17.76)	0.42		NA	NA
>45	4/49 (8.20)	Referent			NA	NA
HIV status						
Negative	31/243 (12.8)	Referent			NA	NA
Positive	5/44 (11.4)	1.27 (0.32–5.14)	0.73		NA	NA
Unknown	0/6 (0.0)	Referent	NA		NA	NA
Nutritional status‡						
Underweight	3/23 (13.0)	0.76 (0.08–6.95)	0.80		NA	NA
Normal weight	21/160 (13.1)	Referent			NA	NA
Overweight	11/107 (10.2)	0.76 (0.23–2.47)	0.65		NA	NA
Underlying illness§						
No	34/285 (11.9)	Referent			NA	NA
Yes	2/8 (25.0)	16.93 (0.61–471.04)	0.09		NA	NA
Crowding¶						
No	8/100 (8.0)	Referent			NA	NA
Yes	28/193 (14.5)	1.84 (0.21–16.42)	0.59		NA	NA
Sleep in same room as index case-patient						
No	24/158 (15.2)	Referent			NA	NA
Yes	12/134 (8.9)	0.71 (0.22–2.26)	0.56		NA	NA

*Bold indicates statistical significance. Variables adjusted for in final model: sex and episode duration. Ct, cycle threshold; HCIR, household cumulative infection risk; IS, insertion sequence; NA, not applicable; PHIRST, Prospective Household cohort study of Influenza, Respiratory Syncytial virus, and other respiratory pathogens community burden and Transmission dynamics in South Africa. †Used as a proxy for bacterial load. ‡Nutritional status is based on a person’s body mass index (BMI). We defined BMI categories as follows: underweight, age <18 y weight for age or BMI <‒2 SDs World Health Organization (WHO) Child Growth Standards; age >18 y BMI <18.5 kg/m^2^; overweight, age <18 y BMI >+1 and ≤+2 SD of the WHO growth standards, age ≥18 y BMI ≥25 and <30kg/m^2^; obese, age <18 y BMI >+2 SD of WHO growth standards, age ≥18 y BMI >30 kg/m^2^. §Defined as self-reported history of asthma, lung disease, heart disease, stroke, spinal cord injury, epilepsy, organ transplant, immunosuppressive therapy, organ transplantation, cancer, liver disease, renal disease, or diabetes. ¶More than 2 persons in a household sleeping in the same room.

## Discussion

In this study population, we found a high incidence of *B. pertussis* and most infected persons were colonized. We detected 118 episodes of *B. pertussis* infection in 107 participants, of which 11 persons had 2 episodes. The overall HCIR was 14%, and 39% of index cases in the household were colonized with *B. pertussis*. The mean duration of PCR-positive *B. pertussis* infection was 12 days. Persons positive for *B. pertussis* were more likely to report symptoms if they were infected for >7 days. Transmission was more likely to occur from male index case-patients and persons who had longer episode duration.

In this community cohort study of healthy persons in South Africa, the incidence of *B. pertussis* was 0.21 cases/100 person-weeks. Incidence in this study was higher than the mean annual incidence risk of 17 cases/100,000 population previously reported in South Africa for persons hospitalized because of pneumonia during 2013–2018 (*9*). This difference was probably caused by the fact that the study conducted by Wolter et al. (*9*) was a cross-sectional study enrolling hospitalized patients who had respiratory illness, whereas our study was a community cohort study enrolling healthy persons. It is difficult to compare our incidence with those of other studies that report incidence data because our study was a community cohort study, whereas other studies focus on populations with *B. pertussis* disease or populations that had outbreaks of *B. pertussis*. Thus, more data describing the incidence of *B. pertussis* among healthy persons over time, and additional longitudinal community data are required for meaningful comparisons to be made. Assessing factors associated with *B. pertussis* incidence, children <5 years of age who did not receive the full schedule of pertussis vaccine for age were more likely to be infected with *B. pertussis* than were vaccinated children. Similarly, other studies in South Africa showed that risk for pertussis and risk for hospitalization caused by pertussis decreased when persons were vaccinated (*9*,*14*).

Data on duration of PCR-positive *B. pertussis* infection among persons are limited. In our study, the average duration of naturally acquired *B. pertussis* infection was 12 days and episode duration was longer for participants with higher bacterial loads, similar to findings for influenza virus infection in the same cohort (*11*). A human challenge study that induced *B. pertussis* colonization in healthy adults showed that *B. pertussis* persisted within the nasopharynx for up to 16 days postinoculation in some of the study participants (*15*). However, it should be noted that in the human challenge study, adults had received vaccination within the previous 5 years and were treated for colonization from day 14. In our study, data on vaccination of persons >5 years of age and treatment were missing, and that information could have potentially affected the duration of *B. pertussis* within the nasopharynx of study participants.

Symptoms associated with *B. pertussis* PCR-positive cases vary from mild, nonspecific respiratory symptoms to pertussis-specific symptoms. In this study, symptomatic *B. pertussis*–infected persons reported nonspecific respiratory symptoms, such as runny nose, cough, fever, or sore throat. For the 2018 subset of symptomatic persons who were investigated for *B. pertussis*–specific symptoms, 60% met the notifiable medical condition pertussis case definition. A study in Spain determining factors influencing the spread of *B. pertussis* in households found that participants of all ages experienced *B. pertussis*–specific signs and symptoms, such as cough lasting >2 weeks, paroxysmal cough, posttussive vomiting, inspiratory stridor, apnea, and fever (*16*). In South Africa, common signs and symptoms reported in children hospitalized with laboratory pertussis were nonspecific cough and fever (*17*).

Evidence suggests that colonization is a major trait of the *B. pertussis* pathogen. In our study, two thirds of persons infected with *B. pertussis* did not report any symptoms. *B. pertussis* incidence data from the United States and the United Kingdom provided evidence of colonization of *B. pertussis* (*18*). de Graaf et al. showed that colonization could be induced by the intranasal inoculation of *B. pertussis* in a human challenge model (*15*). Warfel et al. found that persons previously vaccinated with the acellular pertussis vaccine can become colonized with *B. pertussis* (*19*). There is evidence in the literature supporting colonization of *B. pertussis* in the nasopharynx of persons, which was also observed in our study. In addition to colonization, we found a high proportion of index case-patients transmitting *B. pertussis* to other household contacts. However, it is difficult to draw any conclusions from that result because additional longitudinal community data are required for meaningful comparisons to be made.

The transmission dynamics of *B. pertussis* within households is poorly understood and requires additional research. Within the PHIRST, the overall HCIR was 14%. In 2001, a pertussis outbreak in New South Wales in Australia showed a 22.3% secondary household attack rate (*20*) and increased risk for household transmission when the index case-patient began antimicrobial drug treatment >7 days after onset of symptoms (*20*). Transmission in our study was associated with male sex, and the index case-patient had a long episode duration (>7 days). The association between prolonged episode duration and increased transmission is biologically plausible and has been demonstrated for other pathogens, such as influenza virus (*11*). Previous studies have not documented a difference in transmission between male and female patients (*15*,*20*), so this finding should be further explored.

The first limitation of our study is that it was conducted at only 2 sites in South Africa, and results might not be generalizable to other areas and communities within this country. Over the study period, specimens were collected from each person twice a week for a period of 6–10 months. Because we did not collect samples for a full year and therefore could not accurately estimate the incidence over the period of a year, we presented incidence as per 100 person-weeks. Vaccination status was confirmed only for children <5 years of age, so we could conduct analyses regarding vaccination only for this age group. Symptom data collection was poor for 2016 but improved in 2017 and 2018; therefore, the symptom data presented could be an underestimate. The number of *B. pertussis* cases detected in this study is small, so some statistical analyses were underpowered.

The PHIRST was unique in that it was a community cohort study investigating *B. pertussis* infection among healthy persons. A major strength of the study was the repeated, longitudinal sampling of persons over time according to a specified schedule, irrespective of symptoms. This study method ensured that the data collected over time were robust against sampling bias and enabled us to identify the large proportion of persons who were colonized with *B. pertussis*.

In conclusion, in 2 disparate communities in South Africa, we found a high incidence of *B. pertussis*, and two thirds of cases were colonizations. We have demonstrated that persons who are colonized with *B. pertussis* can transmit this bacteria to other household members and thus represent a source of transmission to susceptible age groups.

Appendix Additional information on incidence and transmission dynamics of *Bordetella pertussis* infection in rural and urban communities, South Africa, 2016‒2018.

## References

[1] Dalby T, Harboe ZB, Krogfelt KA, Dalby T, Harboe ZB, Krogfelt KA. Seroprevalence of pertussis among Danish patients with cough of unknown etiology. Clin Vaccine Immunol. 2010;17:2016–23. 10.1128/CVI.00270-10 20926698 PMC3008190

[2] Fisman DN, Tang P, Hauck T, Richardson S, Drews SJ, Low DE, et al. Pertussis resurgence in Toronto, Canada: a population-based study including test-incidence feedback modeling. BMC Public Health. 2011;11:694. 10.1186/1471-2458-11-694 21899765 PMC3189138

[3] Witt MA, Katz PH, Witt DJ. Unexpectedly limited durability of immunity following acellular pertussis vaccination in preadolescents in a North American outbreak. Clin Infect Dis. 2012;54:1730–5. 10.1093/cid/cis287 22423127

[4] Zouari A, Smaoui H, Brun D, Njamkepo E, Sghaier S, Zouari E, et al. Prevalence of *Bordetella pertussis* and *Bordetella parapertussis* infections in Tunisian hospitalized infants: results of a 4-year prospective study. Diagn Microbiol Infect Dis. 2012;72:303–17. 10.1016/j.diagmicrobio.2012.01.002 22313629

[5] Baxter R, Bartlett J, Rowhani-Rahbar A, Fireman B, Klein NP. Effectiveness of pertussis vaccines for adolescents and adults: case-control study. BMJ. 2013;347(jul17 1):f4249. 10.1136/bmj.f4249 23873919

[6] The National Institute for Communicable Diseases. Category 1 notifiable medical conditions [cited 2022 Jan 30]. https://www.nicd.ac.za

[7] World Health Organization. WHO and UNICEF estimates of immunization coverage: 2019 revision, 2020 [cited 2022 Apr 24]. https://www.who.int/immunization/monitoring surveillance/data/zaf.pdf

[8] Soofie N, Nunes MC, Kgagudi P, van Niekerk N, Makgobo T, Agosti Y, et al. The burden of pertussis hospitalization in HIV-exposed and HIV-unexposed South African infants. Clin Infect Dis. 2016;63(suppl 4):S165–73. 10.1093/cid/ciw545 27838669 PMC5106620

[9] Wolter N, Cohen C, Tempia S, Walaza S, Moosa F, du Plessis M, et al. Epidemiology of pertussis in individuals of all Ages hospitalized with respiratory illness in South Africa, January 2013‒December 2018. Clin Infect Dis. 2021;73:e745–53. 10.1093/cid/ciab089 33530100

[10] National Health Laboratory Service. Increase in pertussis cases in South Africa [cited 2021 Apr 24]. https://www.nhls.ac.za/increase-in-pertussis-cases-in-south-africa

[11] Cohen C, Kleynhans J, Moyes J, McMorrow ML, Treurnicht FK, Hellferscee O, et al.; PHIRST group. Asymptomatic transmission and high community burden of seasonal influenza in an urban and a rural community in South Africa, 2017-18 (PHIRST): a population cohort study. Lancet Glob Health. 2021;9:e863–74. 10.1016/S2214-109X(21)00141-8 34019838 PMC8262603

[12] Cohen C, McMorrow ML, Martinson NA, Kahn K, Treurnicht FK, Moyes J, et al.; PHIRST group. Cohort profile: A Prospective Household cohort study of Influenza, Respiratory syncytial virus and other respiratory pathogens community burden and Transmission dynamics in South Africa, 2016-2018. Influenza Other Respir Viruses. 2021;15:789–803. 10.1111/irv.12881 34296810 PMC8542945

[13] Moosa F, du Plessis M, Wolter N, Carrim M, Cohen C, von Mollendorf C, et al. Challenges and clinical relevance of molecular detection of *Bordetella pertussis* in South Africa. BMC Infect Dis. 2019;19:276. 10.1186/s12879-019-3869-7 30898099 PMC6429695

[14] Muloiwa R, Dube FS, Nicol MP, Hussey GD, Zar HJ. Risk factors for *Bordetella pertussis* disease in hospitalized children. PLoS One. 2020;15:e0240717. 10.1371/journal.pone.0240717 33057415 PMC7561157

[15] de Graaf H, Ibrahim M, Hill AR, Gbesemete D, Vaughan AT, Gorringe A, et al. Controlled human infection with *Bordetella pertussis* induces asymptomatic, immunizing colonization. Clin Infect Dis. 2020;71:403–11. 10.1093/cid/ciz840 31562530 PMC7353841

[16] Godoy P, García-Cenoz M, Toledo D, Carmona G, Caylà JA, Alsedà M, et al.; Transmission of Pertussis in Households Working Group. Factors influencing the spread of pertussis in households: a prospective study, Catalonia and Navarre, Spain, 2012 to 2013. Euro Surveill. 2016;21:1–10. 10.2807/1560-7917.ES.2016.21.45.30393 27918260 PMC5144939

[17] Muloiwa R, Dube FS, Nicol MP, Zar HJ, Hussey GD. Incidence and diagnosis of pertussis in South African children hospitalized with lower respiratory tract infection. Pediatr Infect Dis J. 2016;35:611–6. 10.1097/INF.0000000000001132 26967813

[18] Althouse BM, Scarpino SV. Asymptomatic transmission and the resurgence of *Bordetella pertussis.* BMC Med. 2015;13:146. 10.1186/s12916-015-0382-8 26103968 PMC4482312

[19] Warfel JM, Zimmerman LI, Merkel TJ. Acellular pertussis vaccines protect against disease but fail to prevent infection and transmission in a nonhuman primate model. Proc Natl Acad Sci U S A. 2014;111:787–92. 10.1073/pnas.1314688110 24277828 PMC3896208

[20] Terry JB, Flatley CJ, van den Berg DJ, Morgan GG, Trent M, Turahui JA, et al. A field study of household attack rates and the effectiveness of macrolide antibiotics in reducing household transmission of pertussis. Commun Dis Intell Q Rep. 2015;39:E27–33. 26063095 10.33321/cdi.2015.39.4

