# Supplementary material for: Incidence and Transmission Dynamics of Bordetella pertussis Infection in Rural and Urban Communities, South Africa, 2016‒2018
Source: Emerg Infect Dis. 2023 Feb;29(2):294–303. doi: 10.3201/eid2902.221125 (PMC9881781; doi:10.3201/eid2902.221125)
Supplement: Appendix — Additional information on incidence and transmission dynamics of Bordetella pertussis infection in rural and urban communities, South Africa, 2016‒2018. [file 22-1125-Techapp-s1.pdf]

Article DOI: https://doi.org/10.3201/eid2902.221125

Incidence and Transmission Dynamics of *Bordetella pertussis* Infection in Rural and Urban Communities, South Africa, 2016–2018

Appendix

Appendix Table. Characteristics of 24 persons positive for *Bordetella pertussis* who were retrospectively interviewed after a positive PCR result, PHIRST study, South Africa, 2018*

Case-patient	Age, mo	HIV status	Vaccine status†	Cough	Paroxysmal cough	Inspiratory whoop	Posttussive vomiting	Apnea‡	Pertussis case definition§
1	3	Negative	Incomplete	No	No	No	No	NA	No
2	3	Negative	Full coverage	Yes	Yes	No	No	NA	Yes
3	3	Negative	Incomplete	Yes	Yes	Yes	No	NA	Yes
4	5	Negative	Full coverage	Yes	Yes	Yes	Yes	NA	Yes
5	5	Negative	Full coverage	Yes	No	No	No	NA	No
6	5	Negative	Full coverage	Yes	No	No	No	NA	No
7	8	Negative	NA	Yes	Yes	No	No	NA	Yes
8	8	Negative	NA	Yes	Yes	Yes	No	NA	Yes
9	9	Negative	NA	Yes	No	No	No	NA	No
10	9	Negative	NA	Yes	Yes	Yes	Yes	NA	Yes
11	9	Negative	NA	Yes	No	No	No	NA	No
12	9	Negative	NA	No	No	No	No	NA	No
13	10	Unknown	NA	Yes	No	No	No	NA	No
14	12	Negative	NA	Yes	No	No	No	NA	No
15	12	Negative	NA	Yes	Yes	Yes	No	NA	Yes
16	12	Negative	NA	Yes	No	No	No	NA	No
17	12	Negative	NA	No	No	No	No	NA	No
18	15	Negative	NA	Yes	No	Yes	No	NA	Yes
19	36	Positive	NA	Yes	No	No	No	NA	No
20	37	Positive	NA	Yes	Yes	Yes	No	NA	Yes
21	38	Negative	NA	Yes	Yes	No	No	NA	Yes
22	38	Negative	NA	No	No	No	No	NA	No
23	40	Negative	NA	Yes	Yes	Yes	Yes	NA	Yes
24	59	Positive	NA	Yes	Yes	No	No	NA	Yes

*NA, not applicable.
†Only for children <5 y of age. Vaccine status calculated based on number of doses of vaccine received by age.
‡Apnea in infants <1 y of age.
§Clinical case definition is cough with 1 of the following: paroxysmal cough, inspiratory whoop, posttussive, vomiting, and apnea. A total of 51 persons were positive for *B. pertussis* in 2018.

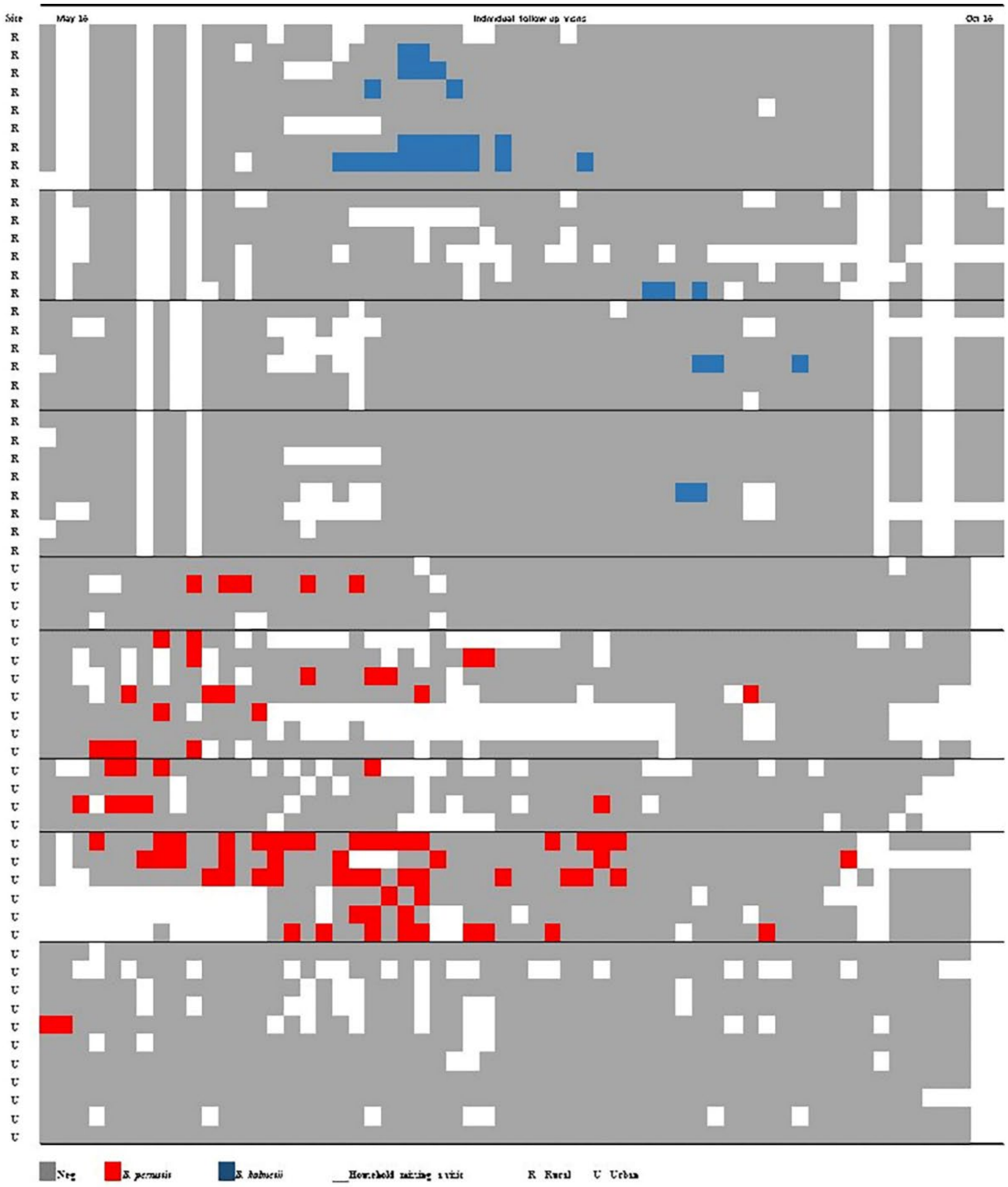

Appendix Figure 1. Community *Bordetella pertussis* and *B. holmesii* infections among study participants (households with cases only) by site, PHIRST study, South Africa, 2016. Columns Indicate individual follow-up visits, and rows indicate individual study participants. R, rural; U, urban.

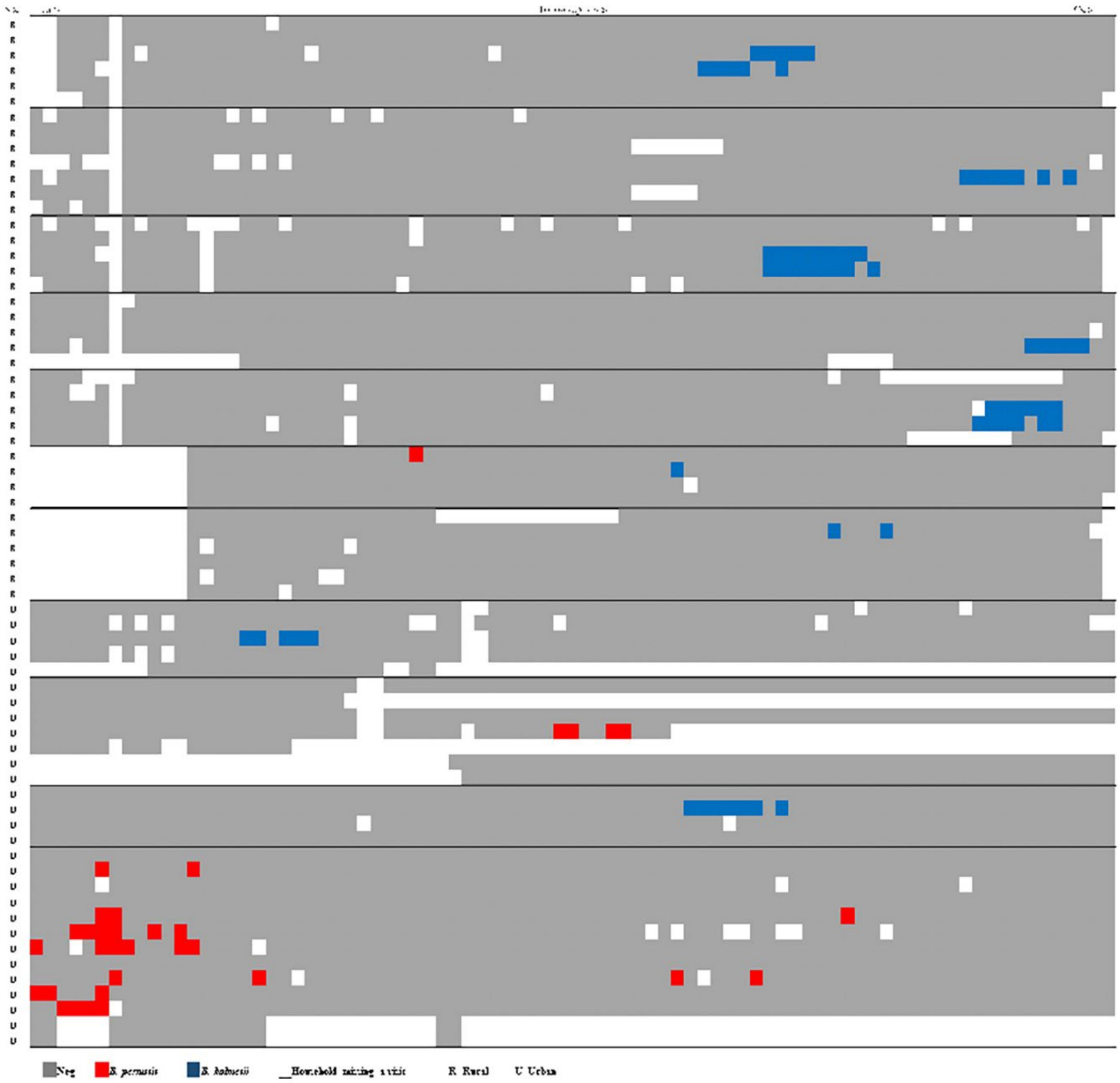

Appendix Figure 2. Community *Bordetella pertussis* and *B. holmesii* infections among study participants (households with cases only) by site, PHIRST study, South Africa, 2017. Columns Indicate individual follow-up visits, and rows indicate individual study participants. R, rural; U, urban.

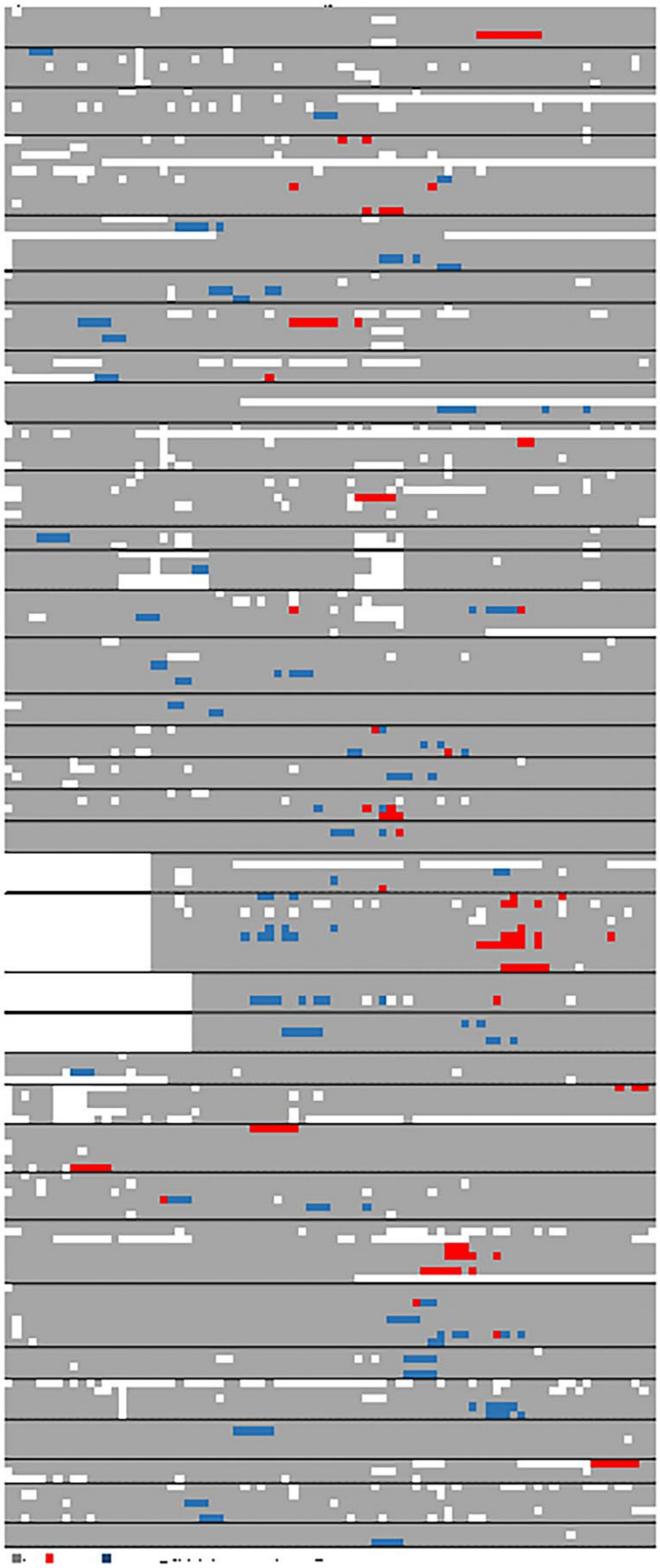

Appendix Figure 3. Community *Bordetella pertussis* and *B. holmesii* infections among study participants (households with cases only) by site, PHIRST study, South Africa, 2018. Columns Indicate individual follow-up visits, and rows indicate individual study participants. R, rural; U, urban.
